# Prevalence of nutrient canals in mandibular anterior intra-oral periapical radiographs (IOPARS) in patients with chronic systemic diseases – a cross-sectional study

**DOI:** 10.25122/jml-2022-0009

**Published:** 2022-05

**Authors:** Shamimul Hasan, Ulfat Jahan, Shazina Saeed, Mandeep Kaur, Virender Gombra, Arpita Rai, Shahnaz Mansoori

**Affiliations:** 1. Department of Oral Medicine and Radiology, Faculty of Dentistry, Jamia Millia Islamia, New Delhi, India; 2. Faculty of Dentistry, Jamia Millia Islamia, New Delhi, India; 3. Amity Institute of Public Health, Amity University, Noida, India

**Keywords:** chronic periodontitis, diabetes mellitus, hypertension, mandibular anterior IOPAR, nutrient canals

## Abstract

Nutrient canals (NCs) are passages containing neurovascular bundles (blood vessels, lymph vessels, and nerves). Best visualized on mandibular anterior intraoral periapical radiographs (IOPAR), their presence is usually associated with underlying pathological such as diabetes mellitus (DM), hypertension (HTN), advanced periodontitis, calcium deficiency, tuberculosis, and disuse atrophy. This study aimed to (a) evaluate NC prevalence in patients with DM, HTN, and chronic periodontitis and (b) correlate the detection of NCs as an important preliminary screening tool for inherent systemic diseases like DM, HTN, and chronic periodontitis and as an investigative clue in age and gender determination. This cross-sectional study was conducted on 200 subjects. Patients with complaints of pain in the mandibular anterior teeth, deep dentinal caries, abrasion, and attrition were subjected to IOPAR of the mandibular anterior teeth region to assess NCs. An increased frequency of NCs in DM (84%), HTN (66%), and periodontitis (52%) with a significant p-value was observed. Most NCs were seen beyond the root apex (72.4%). A notable association between the duration of disease and the presence of NCs in the diabetic and hypertensive cohorts (p-value 0.047 & 0.012, respectively) was observed. However, we could not establish any association between the prevalence of nutrient canals with age and gender. Our study suggested that a higher frequency of NCs on mandibular anterior IOPAR may be employed as an ancillary screening and investigative support in underlying systemic disorders.

## INTRODUCTION

Radiographs serve as an essential investigative tool in dentistry and are largely employed to detect dental caries and sequelae, periodontal diseases, and moderately overt bony changes in the maxilla and mandible. The anatomical structures often exhibit an extensive disparity in the radiographic manifestations and may be a sign of underlying systemic diseases. *e.g*., size and thickness of trabeculae, the pulp chamber and canal size, and the presence of nutrient canals [1]. Nutrient canals are one of the important anatomical landmarks visualized on intraoral periapical radiographs (IOPAR). The first radiographic description of nutrient canals was made by Hirschfield (1923), being regarded as passages containing neurovascular bundles (blood vessels, lymph vessels, and nerves) [2]. They are now assigned names such as interdental canals, circulating canals, and vascular canals [3].

Nutrient canals are mostly visualized on IOPARs of mandibular anterior teeth. Sometimes, they are also visualized in the region of mandibular premolar teeth and maxillary sinus wall [4]. Nutrient canals are usually seen as radiolucent lines of varying thickness, run a vertical course, and are positioned interproximally and inferiorly to the teeth [5, 6]. Existing literature has established a 5% prevalence in the general population, more commonly seen in Black people and males [6]. Some researchers consider nutrient canals as normal anatomical structures, in contrast to others who associate their presence with an underlying pathological state such as advanced periodontitis, diabetes mellitus, hypertension, calcium deficiency, tuberculosis, and disuse atrophy [6, 7].

The presence of nutrient canals may pose certain problems in dental surgical techniques, such as a source of focal bleeding, a potential route of infection spread, and difficulty in attaining adequate anesthesia [8]. They also have a role in evaluating prospective implant sites, as the radiographic occurrence of nutrient canals may suggest a knife-edged alveolar ridge [9]. Few studies suggested a notable association between nutrient canals prevalence and age and gender estimation, thus posing promising diagnostic support in forensic odontology [5, 10, 11].

Our objectives were to:

- Evaluate the prevalence of nutrient canals in patients with chronic systemic disorders (diabetes mellitus (DM), hypertension, and chronic periodontitis) and compare different patient groups;
- Correlate the detection of nutrient canals as a preliminary screening tool for inherent systemic disorders and as an investigative clue in forensic odontology for age and gender determination.

## MATERIAL AND METHODS

A prospective, cross-sectional study was conducted for ten months (June 2019–April 2020) on 800 subjects reporting to the Outpatient Department of Oral Medicine and Radiology, Faculty of Dentistry, Jamia Millia Islamia.

The patient cohort included patients of either gender, in the age group of 20–70 years, with at least one-year history of DM and hypertension (and on medications), clinical signs of chronic periodontitis (bleeding on probing, gingival recession, pocket depth ≥4 mm, plaque and calculus, and tooth mobility). The control cohort included patients with a negative history of DM, hypertension, and other systemic diseases, good oral hygiene, and no clinical signs of periodontitis. Individuals unwilling to participate, subjects less than 20 years of age and more than 70 years of age, history of both DM, hypertension, and other coexisting systemic ailments, pregnant and lactating females, and poor-quality radiographs were excluded from the study.

Patients with pain in the anterior mandibular tooth region, deep proximal caries with dentinal involvement, and attrition were selected for further radiographic evaluation based on the above-mentioned inclusion criteria. The radiographs were taken as part of the treatment protocol and were further analyzed and interpreted for the study. However, no extra radiographs were taken for the study.

The subjects were divided into 4 groups. The 1^st^ group comprised 50 controls with no clinical signs of periodontitis and a negative history of diabetes mellitus and hypertension or any other chronic systemic disease. The 2^nd^ and 3^rd^ groups were a cohort of 50 patients, each with a known history of DM and hypertension for more than a year, respectively. Group 4 consisted of 50 patients with clinical signs of periodontitis (bleeding on probing, pocket depth equal to/more than 4 mm, plaque and calculus, and tooth mobility).

All four groups were further subdivided with attributes related to missing mandibular anterior teeth and level of interdental bone loss (no bone loss, bone loss up to middle/apical 1/3^rd^ of the root).

Patient information (demographics and past medical history) was documented in a study proforma designed for this research. The clinical examination was conducted following infection control measures (facemask, gloves, sterilized mouth mirrors, and probes), under an adequate light source and the patient sitting comfortably on the dental chair in the supine position.

The patients were subjected to an IOPAR of the anterior mandibular teeth region (43–33 region) using a bisecting angle technique with 70 kilo voltages (KVP), 10 ma, and an exposure time of 0.6 sec. Standard adult size (size 2) E-speed films were used and processed manually. The radiographs were taken (following the radiation protection guidelines), dried, and visualized on an X-ray viewer. These radiographs were assessed for the presence, number, and location of nutrient canals in the selected participant cohort using a magnifying glass.

The observations were entered on master charts. Data entries were organized, and statistical evaluation was done using SPSS 20.0 statistical software (Microsoft Corporation Inc., Chicago, IL, USA). A chi-square test was performed to compare different patient groups, and a p-value of <0.05 was considered statistically significant.

## RESULTS

Out of 800 screened subjects, 200 subjects (both males and females) fulfilling the inclusion and exclusion criteria were enrolled in the study. A total of 97 males and 103 females participated in the study. Within the sub-groups, there were 28 males and 22 females in group I, 22 males and 28 females in group II, 21 males and 29 females in group III, and 26 males and 24 in group IV. The results are depicted in Table 1.

**Table 1:** The distribution of nutrient canals among the various groups, sub-groups and gender.

Groups	Subgroups	Total no.	NC +ve	NC -ve	Gender	Total no.	NC +ve	NC -ve	p-value
**Controls**	Missing lower anterior teeth	5	1	4	Male	28	6	22	0.7757
	No interdental bone loss	15	3	12	%		21.4	78.5	
	Interdental bone loss up to middle 1/3^rd^	29	4	25	Female	22	4	18	
	Interdental bone loss up to apical 1/3^rd^	6	2	4	%		18.1	81.8	
**Diabetes Mellitus**	Missing lower anterior teeth	8	8	0	Male	22	20	2	0.2375
	No interdental bone loss	2	2	0	%		90.9	9.1	
	Interdental bone loss up to middle 1/3^rd^	27	22	5	Female	28	22	6	
	Interdental bone loss up to apical 1/3^rd^	21	18	3	%		78.5	21.4	
**Hypertension**	Missing lower anterior teeth	5	3	2	Male	21	16	5	0.1955
	No interdental bone loss	5	3	2	%		76.1	23.8	
	Interdental bone loss up to middle 1/3^rd^	34	23	11	Female	29	17	12	
	Interdental bone loss up to apical 1/3^rd^	11	7	4	%		58.6	41.3	
**Chronic Periodontitis**	Missing lower anterior teeth	12	7	5	Male	26	13	13	0.7682
	No interdental bone loss	2	0	2	%		50	50	
	Interdental bone loss up to middle 1/3^rd^	27	10	17	Female	24	13	11	
	Interdental bone loss up to apical 1/3^rd^	21	15	6	%		54.1	45.8	

**Figure 1:**
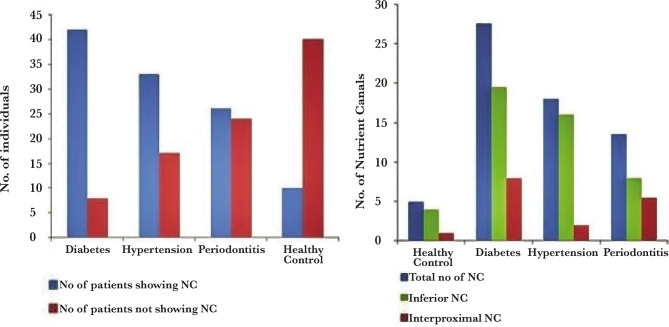
The comparison of different groups regarding the presence/absence and location of nutrient canals.

Among the controls, most individuals (n=4) showed nutrient canals with interdental bone loss up to the middle 1/3^rd^ of the root. The diabetic cohort exhibited nutrient canals with interdental bone loss up to middle and apical 1/3^rd^ (n=22 and n=18, respectively). Also, all diabetic individuals showed the presence of nutrient canals missing lower anterior teeth (n=8). Hypertensive patients (n=23) showed the maximum number of nutrient canals with interdental bone loss up to 1/3^rd^. Patients showing clinical signs of periodontitis exhibited the maximum number of nutrient canals with interdental bone loss up to apical 1/3^rd^ (n=15). The results are shown in Figure 1 and Table 1.

When the presence of nutrient canals was compared among males and females across different groups, there was no predilection for any gender. In the patient cohort with periodontitis, nutrient canals were present among 50% of males and 54.1% of females (p-value=0.768). Among diabetic patients, 90.9% of males and 78.5% of females showed nutrient canals with a p-value of 0.237. The group of patients with hypertension showed that 76.1% of males and 58. 6% of females had nutrient canals (p-value=0.195). Among the healthy controls, a very small percentage of 21.4% and 18.1% of males and females, respectively, had nutrient canals (p-value=0.775). Overall, all the groups showed a non-significant p-value suggesting no association between gender and the presence of nutrient canals across all the groups, as shown in Table 1.

When the four groups were analyzed according to age groups, all four showed non-significant p-values of 0.509, 0.536, 0.482, and 0.090 for healthy controls, diabetes mellitus, hypertension, and periodontitis. Our study did not suggest a possible relationship between age and the prevalence of nutrient canals, as shown in Table 2.

**Table 2:** Distribution of nutrient canals among the various groups and age groups.

S.no	Age (yrs)	Controls (n)	NC +ve n (%)	NC -ve n (%)	p-value	Diabetes (n)	NC +ve n (%)	NC -ve n (%)	p-value	Hypertension (n)	NC +ve n (%)	NC -ve n (%)	p-value	Periodontitis (n)	NC +ve n (%)	NC -ve n (%)	p-value
**1**	21–30	31	5 (16.12%)	26 (83.9%)	0.509	-	-	-	0.5362	6	2 (33.30%)	4 (66.70%)	0.482	10	2 (20%)	8 (80%)	0.090
**2**	31–40	12	1 (8.33%)	11 (91.7%)		15	13 (86.70%)	2 (13.30%)		9	4 (44.40%)	5 (55.60%)		13	9 (69.20%)	4 (30.70%)	
**3**	41–50	7	2 (28.6%)	5 (71.42%)		16	13 (81.30%)	3 (18.70%)		12	7 (58.30%)	5 (41.70%)		11	8 (72.70%)	3 (27.30%)	
**4**	51–60	-	-	-		13	12 (92.30%)	1 (7.70%)		17	12 (70.60%)	5 (29.40%)		11	5 (45.40%)	6 (54.60%)	
**5**	61–70	-	-	-		6	4 (66.70%)	2 (33.30%)		6	4 (66.70%)	2 (33.30%)		5	2 (40%)	3 (60%)	

The presence of nutrient canals and their association with the duration of the disease, especially among individuals with chronic diseases, was significant, with p-values of 0.047 for diabetes mellitus and 0.012 for hypertension, respectively. This finding suggests that the duration of diseases was correlated with the presence of nutrient canals, as shown in Table 3.

**Table 3:** Presence of nutrient canals with respect to the duration of disease.

Duration in yrs	Diabetes	No. of NC	p-value	Hypertension	No. of NC	p-value
**1–5**	25	39	0.04797*	30	32	0.01283*
**6–10**	21	11		16	3	
**11–15**	4	5		4	1	
**16–20**	0	0		0	0	
**More than 20 yrs**	0	0		0	0	
**Total**	50	55		50	36	

* – Significant P-value<0.05.

Further analysis of all the groups with the control cohort revealed a significant result with extremely significant p-values of 0.000004, 0.000003, and 0.00085, respectively, among the diabetes, hypertension, and periodontitis group. Our study revealed an increased frequency of nutrient canals in the disease groups, which is not commonly seen among healthy individuals. The results are shown in Table 4.

**Table 4:** Comparison between groups and controls for the presence of nutrient canals.

Groups	No. of patients showing NC	No. of patients not showing NC	p-value
**Diabetes Mellitus**	42	8	0.000004*
**Control**	10	40	
**Hypertension**	33	17	0.000003*
**Control**	10	40	
**Chronic Periodontitis**	26	24	0.00085*
**Control**	10	40	

* – Significant P-value<0.05.

Our intergroup analysis revealed that the highest number of nutrient canals were seen among diabetes patients, followed by hypertension and chronic periodontitis with a significant p-value of 0.0028, as depicted in Table 5.

**Table 5:** Comparison of inter-groups regarding the presence of nutrient canals.

Groups	No of patients showing NC	No of patients not showing NC	p-value
**Diabetes Mellitus**	42	8	0.0028*
**Hypertension**	33	17	
**Chronic Periodontitis**	26	24	

* – Significant P-value<0.05.

**Figure 2:**
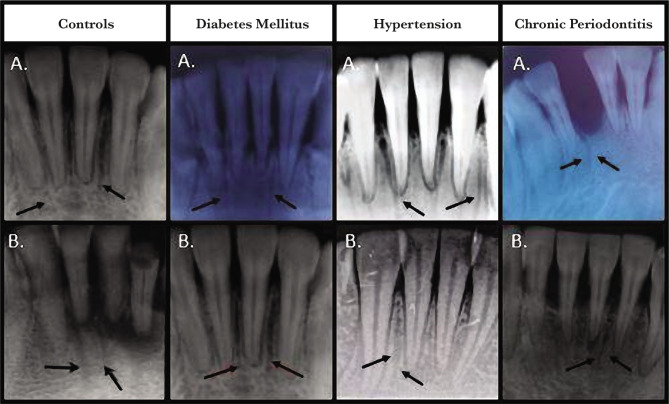
Intraoral periapical radiographs (IOPARs) among various groups. (A) and (B) depicts the radiographic visualization of nutrient canals in control, diabetes, hypertension and chronic periodontitis groups.

Figure 2 (A and B) represents the pictorial representation of the intraoral periapical radiographs (IOPARs) among the various cohort groups.

## DISCUSSION

Indistinguishable anatomical landmarks undergo an extensive disparity in radiographic evaluation [5]. Amongst the radiographic anatomical structures, nutrient canals continue to be one of the most mysterious in their presence/absence. Many studies have been conducted to establish a possible association between the presence of nutrient canals in both the diseased and healthy states [3, 6, 12–14].

Nutrient canals are duct-like channels in the alveolar bone encompassing neurovascular bundles that supply the teeth and adjacent supporting landmarks [15].

Regardless of the small film size and two-dimensional nature, intraoral periapical radiographs (IOPARs) provide excellent delineation of the nutrient canals, where they are visualized as linear radiolucencies, varying in number, size, and location with respect to the tooth roots [16]. Nutrient canals are characteristically visualized in the mandibular anterior teeth radiographs. The most likely reason for this site predilection may be thin alveolar bone with fine and sparse trabecular pattern and lesser superimposition of the anatomical landmarks [2, 12, 17].

Bisecting angle radiographic technique was employed in the present study. Comparable results were seen in other studies [4, 5, 18–20]. Selarka et al. [11], Patel et al. [12], Kaur et al. [16], Castelino et al. [21], and Singh et al. [22] employed the paralleling radiographic techniques. The radiographic technique was not mentioned in the studies by Goodman et al. [23], Reddy et al. [24], and Yustiaputri et al. [25].

In the present study, out of 200 screened subjects, 103 were females (51.7%), and 97 were males (48.5%). When the presence of nutrient canals was compared among males and females across different groups, there was no predilection for any gender. Among the healthy controls, a very small percentage of 21.4% and 18.1% of males and females, respectively, had nutrient canals (p-value=0.775). Among diabetic patients, 90.9% of males and 78.5% of females had nutrient canals (p-value=0.237). Among the hypertensive group, 76.1% of males and 58.6% of females had nutrient canals (p-value=0.195). In the patient cohort with periodontitis, nutrient canals were present among 50% of males and 54.1% of females (p-value=0.768). Overall, all the groups showed a non-significant p-value suggesting no association between gender and the frequency of nutrient canals among all groups.

Numerous studies yielded a variable correlation between gender and nutrient canals. For example, a few studies reported a higher frequency of nutrient canals in females [14, 23, 26], whereas others reported a higher frequency in males [27, 28]. However, other studies failed to demonstrate any relationship between gender and nutrient canals [7, 12].

Nutrient canals are regarded as normal anatomical structures, which are radiographically visualized only in 5% of healthy individuals [21]. An increased frequency of nutrient canals in controls was noted in the studies conducted by Lovette (92%) [3], Bali et al. (60%) [7], Selarka et al. (35%) [11], Patel et al. (42.5%) [12], Kaur et al. (23%) [16], and Kaul et al. (57.5%) [27]. Pandarinath et al. [29] demonstrated a significantly lower prevalence of nutrient canals (6%). However, in our study, 20% of the healthy controls demonstrated nutrient canals.

Our study showed that nutrient canals are most prevalent in diabetic patients (84%). Comparable results were documented in other reports [1, 11, 12, 16, 24, 28, 30]. On further comparing the diabetic group with the control cohort for the occurrence of nutrient canals, statistically significant results with a significant p-value of 0.000004 were seen. This increased prevalence of nutrient canals is in coherence with other studies [13, 14, 23, 26]. Bali et al. [7] demonstrated that nutrient canals were visualized in 76% of diabetic subjects. However, their p-value was non-significant.

Isselbacher et al. postulated the hypothesis explaining the higher prevalence of nutrient canals in diabetic subjects [31]. Insulin deficiency in diabetic subjects may have a mitogenic effect on the endothelial cells, resulting in the development of collateral vessels. Atherosclerotic alterations may also occur, causing narrowing of the blood vessel lumen. Thus, the development of collateral vessels functions as a redemptive mode leading to a higher occurrence of nutrient canals in diabetic individuals [31].

Our study did not show any association between age and the presence of nutrient canals in the diabetic subjects (p-value=0.5362). Comparable results were seen in Bali et al. study [7]. However, Patel et al. [12] reported a higher occurrence of nutrient canals in the 25–55-year age range, with a considerable decrease during the 55–65-year age group.

In the diabetic group, 90.9% of males and 78.5% of females had nutrient canals. However, the p-value was not significant (0.2375). This was in coherence with the studies by Bali et al. [7], Patel et al. [12], and Kaur et al. [16], which did not report an association between the prevalence of nutrient canals and gender.

81.25% of patients in the diabetic group demonstrated a moderate-severe bone loss in our study. A similar presentation was noted in the study conducted by Bali et al. [7], where 81% of diabetic patients had poor oral hygiene/periodontitis.

In the present study, all 8 diabetic patients with missing mandibular anterior teeth presented with radiographically demonstrable nutrient canals. Bali et al. [7] and Kishi et al. [13] reported a higher prevalence of nutrient canals in the edentulous group. This relationship may be linked with alveolar bone resorption following tooth loss and thickening of the remaining alveolar bone.

The reported prevalence of nutrient canals in hypertensive subjects was 66%, in contrast to the 20% prevalence in the healthy control group, with a highly significant p-value of 0.000003. These findings agree with other studies [1, 5, 12, 14, 16, 19, 26]. However, no significant correlation between nutrient canal prevalence and hypertension was reported by Yilmaz et al. [15], Jaju et al. [17], and Esfani et al. [18].

Haslett et al. [32] postulated the hypothesis explaining an increased frequency of nutrient canals in hypertensive patients. Arteriolar dilatation, vessel wall hypertrophy and hyperplasia, and arteriosclerosis are the primary pathophysiological alterations in hypertension. In arteriosclerosis, arterial wall thickening along with luminal constriction takes place. These changes are responsible for the formation of new collateral vessels, resulting in a higher frequency of nutrient canals in hypertensive subjects.

In the hypertensive patients, 76.1% of males and 58.6% of females presented nutrient canals. However, the p-value was not significant (0.1955). Comparable results were seen in Patni et al. [5], Bali et al. [7], Patel et al. [12], Kaur et al. [16], Esfani et al. [18], and Reddy et al. [24] studies. Higher frequency of nutrient canals in hypertensive males and females were reported in the studies by Patsakas et al. [28] and Mani et al., respectively [1].

The present study did not show any association between age and frequency of nutrient canals in the hypertensive subjects (p-value=0.482). Few studies reported that the incidence of nutrient canals increased with increasing age [5, 7, 12–14, 18, 33].

In the present study, 67% of hypertensive patients showed a maximum number of nutrient canals with interdental bone loss up to the middle 1/3^rd^. These findings are in coherence with the studies by Bilge et al. [14] and Patsakas et al. [28], which reported a higher frequency of nutrient canals in hypertensive subjects with moderate bone loss (70.0% and 58.3%), respectively. However, an increased occurrence of nutrient canals in hypertensive subjects with severe bone loss (75.8%) was reported in the study by Kumar et al. [20].

Our study did not show marked variation in the frequency of nutrient canals between hypertensive and non-hypertensive edentulous subjects. Similar findings were reported by Kumar et al. [20].

The reported prevalence of nutrient canals in periodontitis patients was 52%, in contrast to the 20% prevalence in the healthy controls (p-value=0.00085). This agrees with Selarka et al. [11], and Pandarinath et al. [29] studies, which showed that the frequency of nutrient canals in the periodontitis group was 54% and 48.8%, respectively.

In our study, most periodontitis patients with severe interdental bone loss (71.2%) showed the radiographic presence of nutrient canals. Similar findings were reported by Selarka et al. [11], Patel et al. [12], Kishi et al. [13], and Castelino et al. [21]. The hypothesis states that the level of bone resorption is directly related to the occurrence and number of nutrient canals (higher bone resorption is associated with increased prevalence of nutrient canals). Advanced periodontitis results in sclerotic alterations in the trabecular pattern of bone. Also, the radiographic ability to record nutrient canals increases in the thinner bony structures [24].

In the patient cohort with periodontitis, nutrient canals were present among 50% of males and 54.1% of females (p-value=0.768). According to Pandarinath et al. study [29], there were no statistically significant differences in the prevalence of nutrient canals in males and females. However, highly significant differences were noted in further comparison with the control group (7% and 5.2% for males and females, respectively).

Our study showed a predilection for the periapical location of nutrient canals (74.2% of nutrient canals were located beyond the root apex). The results confirmed the findings of Reddy et al. study [24], which reported a higher prevalence of nutrient canals extending beyond the root apex. This periapex location of nutrient canals confirms the well-recognized anatomic depiction of vascular supply to the mandibular anterior region [4, 13, 34–36]. However, Kaur et al. [16] reported that most canals were located between the roots (interproximal).

A highly notable association was observed with disease duration, with a p-value of 0.047 and 0.012 for the diabetic and hypertensive cohorts. This contrasted with the findings of Kaur et al. [16], who did not demonstrate any correlation between the duration of the disease and systemic diseases (diabetes and hypertension).

Patients with chronic hypertension undergo calcification of blood vessels (terminal stage of arteriosclerosis), a probable explanation for an abrupt decline in the occurrence of nutrient canals in the long-standing disease states. Similar arteriosclerotic changes are also seen in the elderly, diabetic, and periodontitis patients [24].

Hence, it is imperative to distinguish nutrient canals in intra-oral periapical radiographs as they may be investigative support for an underlying systemic disorder. However, a meticulous clinical evaluation and a detailed history are required to establish the underlying systemic disorder [37].

The present study was conducted at a single center. Thus, the results may not be representative of the entire population group. Further multicentric studies involving a larger population must be conducted for more precise results.

## CONCLUSION

The radiographic presence of nutrient canals has always been controversial, with some investigators considering them as normal anatomic structures and others as pathologic entities associated with DM, hypertension, and periodontitis. Since IOPARs aid in identifying minute discrepancies in the presentation, number, and position of nutrient canals, the radiographic appearance of nutrient canals may raise a suspicion about the underlying undiagnosed systemic ailment such as DM and hypertension.

The present radiographic study demonstrated an increased frequency of nutrient canals in DM, hypertension, and periodontitis, with a significant p-value. Thus, a higher frequency of nutrient canals on mandibular anterior IOPAR may be employed as an ancillary screening and investigative aid in these systemic disorders. The study also revealed that most nutrient canals were seen beyond the root apex and revealed a noteworthy relationship between the duration of disease and the presence of nutrient canals. However, the study could not establish any relationship between the prevalence of nutrient canals with age and gender.
